# Real-World Data of Triplet Combination of Trastuzumab, Lapatinib, and Chemotherapy in HER2-Positive Metastatic Breast Cancer: A Multicenter Retrospective Study

**DOI:** 10.3389/fonc.2020.00271

**Published:** 2020-03-03

**Authors:** Yi Li, Chengcheng Gong, Qianyi Lu, Zhaochun Zhou, Ting Luo, Wei Li, Gang Li, Rui Ge, Fei Xu, Biyun Wang

**Affiliations:** ^1^Department of Medical Oncology, Department of Oncology, Fudan University Shanghai Cancer Center, Shanghai Medical College, Fudan University, Shanghai, China; ^2^The State Key Laboratory of Oncology in South China, Sun Yat-sen University Cancer Center, Collaborative Innovation Center for Cancer Medicine, Guangzhou, China; ^3^Huangpu Branch of Shanghai Ninth People's Hospital, Shanghai, China; ^4^Department of Head, Neck and Mammary Gland Oncology, Cancer Center, West China Hospital, Sichuan University, Chengdu, China; ^5^Department of Medical Oncology, Jiangsu Province Hospital, Nanjing, China; ^6^Minhang Branch, Fudan University Shanghai Cancer Center, Shanghai, China; ^7^Huadong Hospital Affiliated to Fudan University, Shanghai, China

**Keywords:** human epidermal growth factor receptor 2 positive, metastatic breast cancer, trastuzumab, lapatinib, chemotherapy

## Abstract

**Introduction:** Combination of trastuzumab (T) and lapatinib (L) has been showed to significantly improve the prognosis of HER2+ heavily pretreated metastatic breast cancer (MBC). Whether TL combined chemotherapy (TLC) can further improve the efficacy in HER2+ MBC remains to be further studied. The aim of the study was to report the first real-world data of TLC in HER2+ MBC, including the efficacy, safety and treatment patterns.

**Methods:** Patients with HER2+ MBC treated with TLC in 5 institutions of China from September 2013 to July 2019 were included. Progression free survival (PFS), objective response rate (ORR), overall survival (OS), toxicity profile and treatment pattern were reported.

**Results:** A total of 285 patients were included. 88.8% were exposed to trastuzumab and 49.2% received 2 or more lines of systematic therapy before TLC previously. The most common chemotherapy regimens combined with TL were capecitabine (40.7%) and vinorelbine (21.4%) and almost 1/3 received maintenance treatment after TLC. Median PFS was 10.9 months while patients received TLC as first line treatment showed longest median PFS of 20.7 months. Patients pretreated with trastuzumab showed a median PFS of 10.2 months. In patients who pretreated with trastuzumab, the continuation of trastuzumab on the basis of standard lapatinib plus capecitabine had a median PFS of 11.3 months. TL combined with capecitabine or vinorelbine showed no significant difference in median PFS, though TL combined with capecitabine had numerically prolongation (11.4 vs. 8.5 months, *p* = 0.231). Patients had brain metastasis (BM) also showed a median PFS (intracranial and extracranial lesions considered) of 10.6 months. Lines of systematic metastatic treatment was an independent predictive factor of PFS. The median OS was not reached. Two hundred and seventy seven patients were included in ORR analysis. ORR was 42.6%. Toxicities of triplet combinations were tolerable and the most common grade 3 and 4 adverse events were neutropenia (16.8%).

**Conclusions:** TLC demonstrated promising effects and tolerable safety in HER2+MBC, even in patients with BM, providing a theoretical basis for clinical practice.

**Clinical Trial Registration:** ClinicalTrials.gov, Identifier: NCT04001634.

## Introduction

Breast cancer (BC) remains the most common cancer and the leading cause of death in women worldwide (1). About 15–20% of all breast cancers are human epidermal growth factor receptor-2 (HER-2)-positive, which used to be considered as an aggressive phenotype with poor prognosis until the development of anti-HER2 targeted therapy (2).

Trastuzumab (T), a humanized monoclonal antibody that targets the extracellular domain of HER2, significantly improves the progression free survival (PFS) and overall survival (OS) for patients with HER2+ metastatic breast cancer (MBC) (3). However, not all patients equally benefit from trastuzumab therapy, and drug resistance inevitably happens. Lapatinib, a small molecule tyrosine kinase inhibitor (TKI) with intracellular activity against HER2 and epidermal growth factor receptor, has a different mechanism of action than trastuzumab (4, 5). In EGF100151 trial, “patients with HER2+ MBC who had prior exposure to trastuzumab, treatment with lapatinib plus capecitabine (LX) resulted in statistically improved PFS compared with capecitabine alone (hazard ratio [HR], 95% confidence interval [CI], 0.34–0.71; *p* < 0.01)” (6). The combination of LX has become the standard second-line treatment regimen after progression of trastuzumab, however, several studies indicated that continuation of trastuzumab after progression on it was still effective (7, 8). Thus, whether the addition of trastuzumab on the basis of LX can further improve the efficacy is worthy of further study.

Due to the fact that trastuzumab and lapatinib have different HER2 signaling targeting domains and partially non-overlapping mechanisms of action, we have reason to believe that the combination treatment with trastuzumab and lapatinib (TL) should be superior to monotherapy (9). The phase III EGF104900 study proved that in heavily pretreated HER2+ MBC, TL had significant prolonged PFS (11.1 vs. 8.1 weeks, *p* = 0.008,) and OS (14 vs. 9.5 months; *p* = 0.026), as compared with L alone, with a good safety profile (10, 11). The CELEOPATRA study (12) showed that dual blockages of trastuzumab and pertuzumab plus docetaxel significantly prolong the median PFS and median OS than trastuzumab plus docetaxel in the first-line treatment of HER2+ MBC. Thus, trastuzumab plus pertuzumab and taxanes have become the standard first-line treatment for HER2+ MBC. Under the circumstance, whether TL combined chemotherapy (TLC) can further improve the efficacy in HER2+ MBC especially in the first-line setting remains to be further studied.

An Ib phase study (NCT00251433) assessed “the safety, tolerability, and optimally tolerated regimen (OTR)” of TL plus docetaxel as first-line treatment in HER2+ MBC (13). The results show that TL plus docetaxel is a feasible and well-tolerated regimen of untreated HER2+ MBC with preliminary overall response rate (ORR) is 64%. “Two lapatinib/docetaxel OTR doses were recommended (1,250 mg/75 mg/m^2^; 1,000 mg/100 mg/m^2^)” (13). Also, an open-label safety study explored the safety of the TL plus paclitaxel in first-line HER2+ MBC. In the triplet combinations, “750 mg/d lapatinib had the lowest incidence of diarrhea and the ORR was 75%” (14). Another small retrospective study evaluated the efficacy of TL combined capecitabine in the treatment of HER2+ MBC who experienced progression on trastuzumab. Only five patients were enrolled in the study, 3 of them with brain metastasis(BM) (15). After a median follow-up of 15 months, 4 patients were still receiving treatment without progression and the ORR was 60% which indicated that the TL combined capecitabine is effective with well-tolerable in the treatment of HER2+ MBC and worth further explored (15).

Due to drug availability and other reasons, the choice of anti-HER2 drugs in China is quite less. Therefore, in our clinical practice, TLC has become one of the commonly used therapeutic schemes, but the efficacy and safety of TLC is still lack of large-scale clinical research, especially in metastatic setting.

The aim of this study is to evaluate the efficacy and safety of TLC in real-world HER2+ MBC and to provide a theoretical basis for clinical practice. Also, the study can descript treatment pattern of this regimen in real-world. To our knowledge, this is the first report of real-world data of TLC.

## Methods

### Subjects and Study Design

This is a retrospective, multicenter study which included patients with HER2+ MBC treated with TLC at 5 medical institutions, including Fudan University Shanghai Cancer Center, Sun Yat-sen University Cancer Center, West China Hospital Sichuan University, Jiangsu Province Hospital and Minhang Branch of Fudan University Shanghai Cancer Center from September 2013 to July 2019. The Ethics Committee and Institutional Review Board of Fudan University Shanghai Cancer Center approved this study. All investigations were conducted in accordance with the Declaration of Helsinki. Our research is registered at clinicaltrials.gov (NCT04001634).

### Patients

The study included patients with the eligibility criteria: (1) Female patients age ≥ 18 years with “histologically or cytologically confirmed MBC with documentation of HER2 overexpression (i.e., immunohistochemistry 3 + and/or fluorescent *in situ* hybridization-positive by local assessment)” (2) Patients received trastuzumab 6 mg/kg weekly (after the initial 8 mg/kg loading dose) plus lapatinib (750 mg−1,250 mg/day) plus chemotherapy regimen (by physicians' choice), starting from September 2013 to July 2019 in five hospitals mentioned above. (3) Patients had complete medical records.

All data were retrospectively collected from medical records and laboratory results of individual institutions and administered by Fudan University Shanghai Cancer Center.

### Treatment and Dose Modification

Patients were prescribed with TLC in clinical practice. The combination therapy with cytotoxic drugs, and the starting dose, dose modification and treatment discontinuation of TLC were determined by physicians' choice based on previous clinical trials results, general health status and willing of patients.

### Outcomes

PFS was selected as the primary outcome, which was defined as the time from initiating TLC to date of tumor progression or death of any cause. The second outcome measures included ORR, OS, safety, and treatment pattern of TLC. ORR was defined as the percentage of patients with complete response (CR) or partial response (PR). OS was defined as the time period from initial treatment of TLC to death or last follow-up. Adverse events (AEs) were retrospectively collected based on patients' laboratory tests results and medical records.

Tumor response assessments were accessed based on Response Evaluation Criteria in Solid Tumors (RECIST) 1.1 criteria by CT, MRI and physical examination. ALL AEs were graded by the National Cancer Institute Common Terminology Criteria for Adverse Events (CTCAE, 4.03).

### Statistical Analysis

Clinicopathologic characteristics was presented as median (range) or number of patients (percentage).

PFS and OS were estimated by the Kaplan- Meier method and the hazard ratios (HRs) and corresponding 95% confidence intervals (CIs) were estimated using the Cox proportional hazard model. Exploratory univariate analyses were performed with the log- rank test using the following variables: age, disease-free interval (DFI), number of metastatic sites, visceral metastases, lines of metastatic systematic therapy of TLC, trastuzumab resistance status and prior lapatinib treatment.

“Trastuzumab resistance is defined as progression at first radiological reassessment at 8–12 weeks or within 3 months after first-line trastuzumab with or without chemotherapy in the metastatic setting or new recurrences diagnosed during or within 12 months after adjuvant trastuzumab” (16). “Trastuzumab refractoriness is defined as disease progression after two or more lines of trastuzumab-containing regimens that initially achieved disease response or stabilization at first radiological assessment” (16).

Maintenance treatment was defined as patients who have received disease control (including complete remission, partial remission, and stabilization of the disease) after initial TLC (usually 6 to 8 cycles) then switched to another regimen until disease progression.

Cox multivariate models were performed based on the univariate analyses results. All expressed *p-*values and CIs were two tailed. A *P* < 0.05 was considered statistically significant. All statistical analyses were carried out with Statistical Package for the Social Sciences Software (SPSS) version 24.0.

## Results

### Baseline Characteristics

A total of 285 HER2+ MBC treated with TLC between September 2013 and July 2019 in 5 situations were included. Baseline characteristics were shown in Table 1. The median age of patients at diagnosis was 50 (range 36–87) years. Sixty nine patients were *de novo* stage IV breast cancer (24.2%). 41.1% patients had more than 3 metastatic sites. The 3 most common metastatic sites were bone (41.8%), lung (39.3%) and liver (37.5%). 73.3% patients had visceral metastases. In addition, 69 (24.2%) patients had BM. Majority of patients had been exposed to anti-HER2 therapy, with 88.8% patients exposed to trastuzumab and 18.2% exposed to lapatinib. 49.2% patients received 2 or more lines of systematic therapy before TLC, representing a heavily pre-treated group. Thus, it can be seen that in real-world setting, patients receiving TLC more likely to be heavily pretreated.

**Table 1:** Patient characteristics at baseline.

**Characteristics**	**Number of patients (%) (*N* = 285)**
Median age (years, range)	50 (26–87)
**ECOG PERFORMANCE-STATUS SCORE**	
0	78 (27.3)
1	193 (67.7)
2	14 (5.0)
**HORMONE RECEPTOR STATUS**	
HR positive	115 (40.4)
HR negative	170 (59.6)
**DISEASE-FREE INTERVAL**	
Primary metastatic	69 (24.2)
DFI ≤ 1year	71 (25.0)
DFI > 1year	145 (50.9)
**METASTATIC SITES**	
Lung	112 (39.3)
Liver	107 (37.5)
Bone	119 (41.8)
Brain	69 (24.2)
**NUMBER OF METASTATIC SITES**	
1	77 (27.0)
2	91 (32.0)
≥3	117 (41.1)
**VISCERAL METASTASES**	
Yes	209 (73.3)
No	76 (26.7)
**LINES OF ADVANCED SYSTEMATIC THERAPY OF TLC**	
1	65 (22.8)
2	80 (28.1)
≥3	140 (49.1)
**TRASTUZUMAB RESISTANCE STATUS**	
Resistance	118 (41.4)
Refractoriness	133 (46.7)
Unknown	28 (10.0)
**PRIOR HER2-TARGETED THERAPY**	
Trastuzumab	253 (88.8)
Lapatinib	52 (18.2)
T-DM1	15 (5.3)
Pertuzumab	2 (0.7)

*HR, Hormone receptor; ECOG, Eastern Cooperative Oncology Group; DFI, Disease free interval; TLC, trastuzumab+ lapatinib+ chemotherapy*.

Sixty five patients received TLC as their first-line treatment and their baseline characteristics were shown in Supplemental Table 1. In addition, 110 patients received TL plus capecitabine after progressing on trastuzumab and their baseline characteristics were shown in Supplemental Table 2.

### Treatment Administration

Treatment administration was shown in Table 2. The most common starting chemotherapy regimens combined with TL were capecitabine (40.7%), and vinorelbine (21.4%). Other combined regimens also included paclitaxel, paclitaxel plus carboplatin, gemcitabine, docetaxel, vinorelbine plus capecitabine, etc. Ninety three patients received maintenance treatment after initial TLC due to the consideration of patients' general health status and willing of patients. The most common maintenance treatment was TL alone (23.7%), TL combined capecitabine (21.5%), T plus one chemotherapy (19.4%), L plus one chemotherapy (19.4%), etc. Lapatinib was initially prescribed at the dose of 1,250 mg/day in nearly half of the patients, 1,000 mg/day in 99 patients (34.7%), 750 mg/day in 37 patients (13.0%) and at 500 mg/day only in 8 patients (2.8%). Totally 56 (19.6%) patients experienced dose reduction of lapatinib while 6 (0.2%) patients experienced dose escalation due to AEs. 30 (10.5%) patients interrupted the lapatinib treatment. Besides, 53 (18.6%) patients experienced dose reduction of chemotherapy. 16 (5.7%) patients discontinued lapatinib treatment permanently due to AEs. One patient discontinued trastuzumab because left ventricular ejection fraction (LVEF) decrease of 10% relative to baseline.

**Table 2:** Treatment administration.

**TLC treatment**	**Number of patients (%) (*N* = 285)**
**STARTING REGIMENS**	
TL+ capecitabine	116 (40.7)
TL+ vinorelbine	61 (21.4)
TL+ paclitaxel	33 (11.6)
TL+ paclitaxel+ carboplatin	17 (6.0)
TL+ gemcitabine	14 (5.0)
TL+ docetaxel	13 (4.6)
TL+ vinorelbine + capecitabine	7 (2.5)
Other	24 (8.4)
Maintenance treatment regimen	93 (33.0)
TL	22 (23.7)
TL+ capecitabine	20 (21.5)
T+ CT	18 (19.4)
L+ CT	18 (19.4)
TL+ endocrine therapy	7 (7.5)
TL+ another CT	5 (5.4)
T	3 (3.2)
**LAPATINIB**	
**Starting dosage (mg/day)**	
1,250	141 (49.5)
1,000	99 (34.7)
750	37 (13.0)
500	8 (2.8)
**Dose reduction (mg/day)**	
1,250 → 1,000	27 (9.5)
1,250 → 1,000 → 750	4 (1.4)
1,000 → 750	17 (6.0)
1,000 → 750 → 500	4 (3.5)
1,000 → 750 → 1000	1 (0.4)
750 → 500	3 (1.1)
**Dose escalation (mg/day)**	
500 → 750 → 1,000	1 (0.4)
1,000 → 1,200	5 (1.8)
Interruption of lapatinib treatment	30 (10.5)
Lapatinib treatment discontinuation due to AEs	16 (5.7)
**CHEMOTHERAPY**	
**Dose reduction**	
Yes	53 (18.6)
No	232 (81.4)

*T, trastuzumab; L, lapatinib; C; chemotherapy; AEs, adverse events*.

### Efficacy

**Figure 1:**
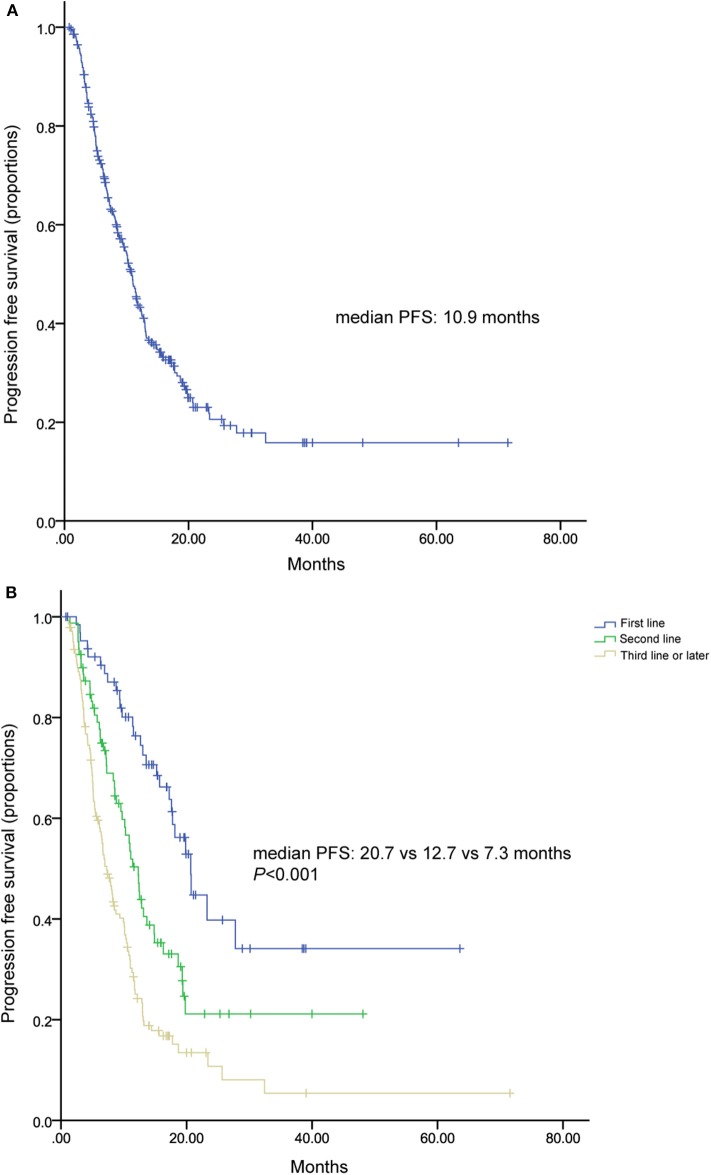
Kaplan-Meier curve of progression free survival **(A)** for all patients and patients stratified by treatment lines **(B)**.

**Figure 2:**
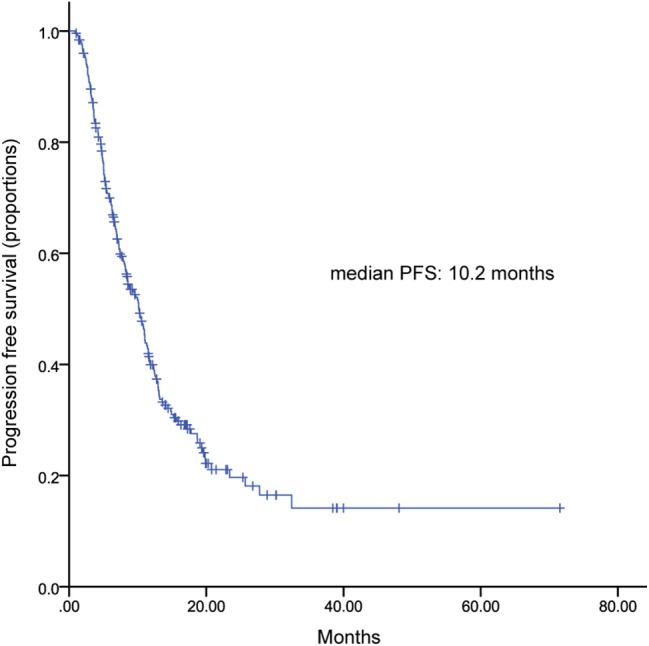
Kaplan-Meier curve of progression free survival for patients pretreated with trastuzumab.

All of patients were included in PFS analysis. At a median follow-up of 16 months, 189 patients experienced progressive disease, resulted in a median PFS of 10.9 (9.67–12.07) months (Figure 1A). Patients received TLC as their first, second, third, and later lines of metastatic treatment had a median PFS of 20.7 (17.14–24.19), 12.3 (10.56–14.04), 7.3 (5.9–8.6) months, respectively (Figure 1B). Two hundred and fifty two patients pretreated with trastuzumab showed a median PFS of 10.2 (8.7–11.7) months (Figure 2).

**Figure 3:**
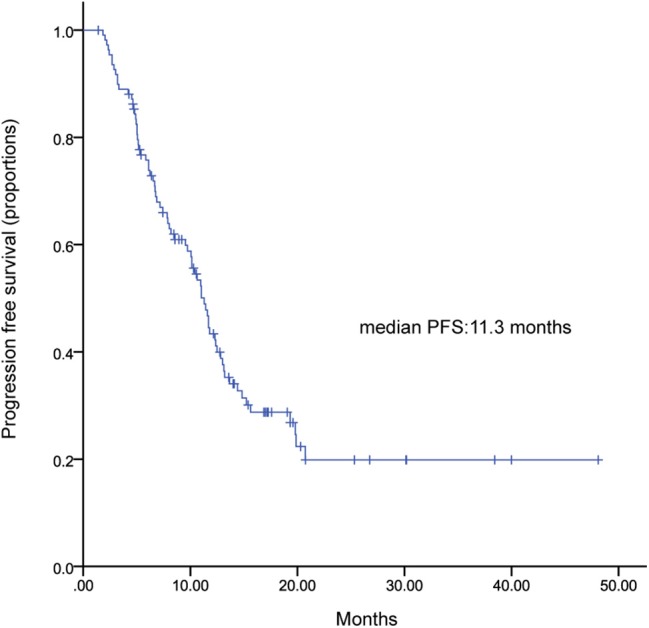
Kaplan-Meier curve of progression free survival for patients received TLX after progressing on trastuzumab.

**Figure 4:**
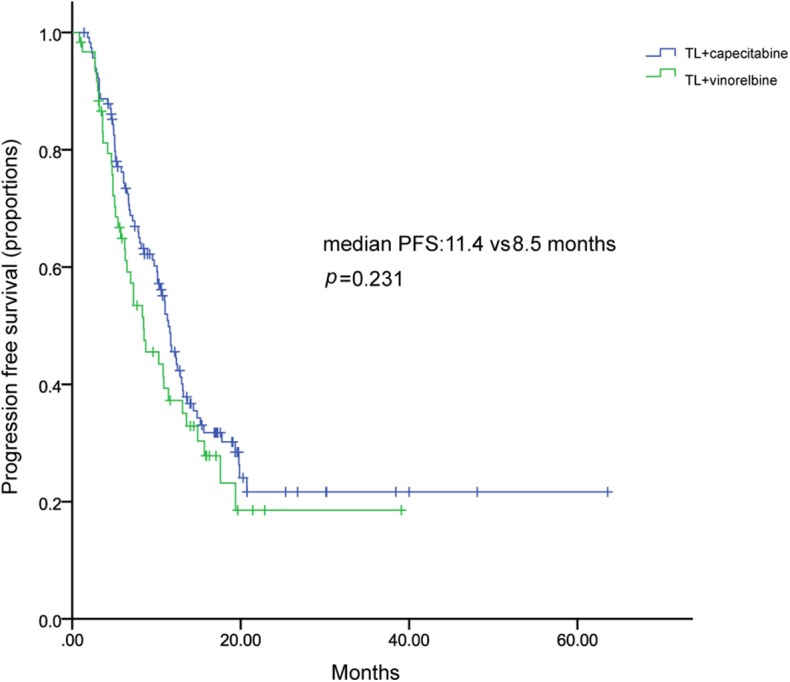
Kaplan–Meier curve of progression free survival of patients treated with TL plus capecitabine or vinorelbine.

**Figure 5:**
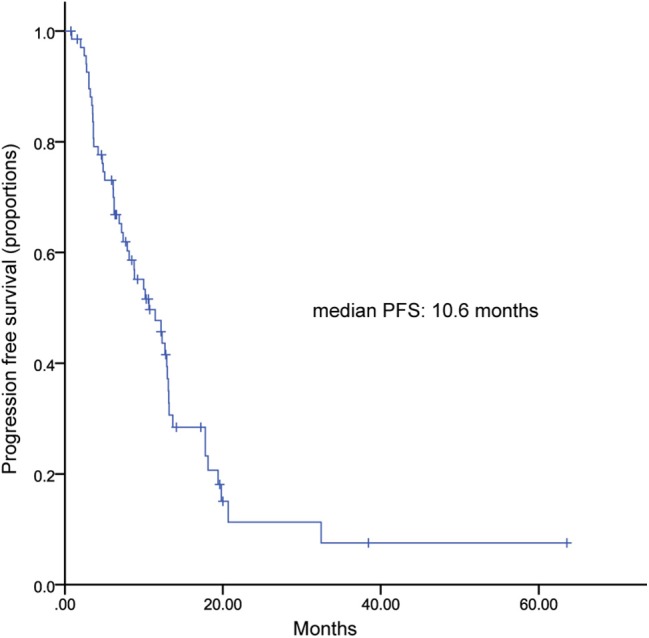
Kaplan-Meier curve of progression free survival in patients with brain metastasis.

One hundred and ten patients received TL plus capecitabine after progressing on trastuzumab, resulting a median PFS of 11.3 months (95% CI, 9.9–12.7) (Figure 3). TL combined with capecitabine or vinorelbine were the most common TLC regimen. The median PFS was not statistically significant between the two regimens while patients received TL plus capecitabine had a numerically longer PFS than those received TL plus vinorelbine (11.4 vs. 8.5 months; HR, 0.791; 95% CI, 0.538–1.162; *p* = 0.231) (Figure 4). Patients with BM also showed a median PFS (intracranial and extracranial lesions considered) of 10.6 (6.84–11.43) months (Figure 5). OS data were not mature at the time of this report. A total of 277 patients were included in ORR analysis, with 8 patients were excluded because of lack of measurable lesions (Table 3). Two patients (0.7%) achieved CR, 116 patients (41.9%) had PR, resulted in an ORR of 42.6%.

**Table 3:** Objective response rate in all patients.

**Response**	**Number of patients (%) (*N* = 277)**
**BEST RESPONSE**	
Complete response	2 (0.7)
Partial response	116 (41.9)
Stable disease	128 (46.2)
Progressive disease	25 (9.0)
NA	6 (2.2)
ORR	118 (42.6)

*NA, not available; ORR, objective response rate*.

**Figure 6:**
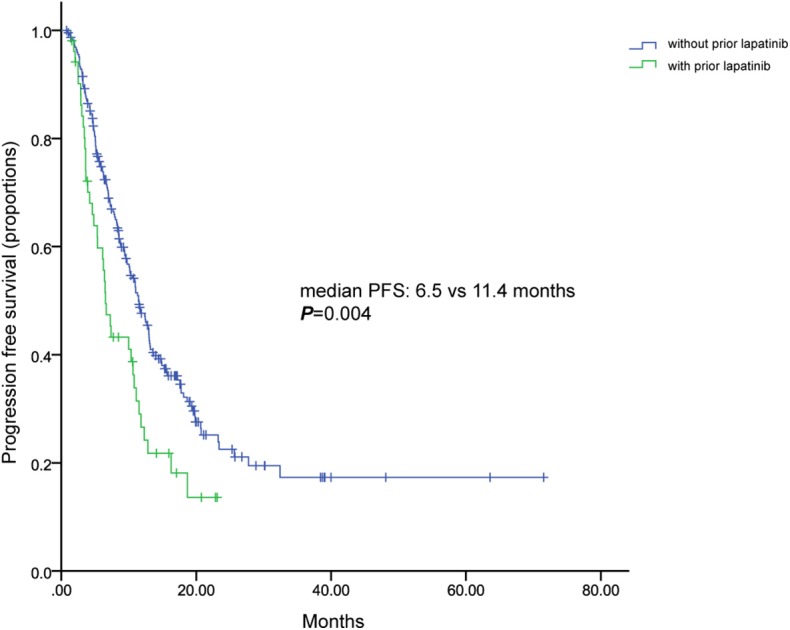
Kaplan-Meier curve of progression free survival for patients with and without previous lapatinib.

Univariate analysis indicated that number of metastatic sites (≤2 vs. >2), types of metastasis (visceral vs. non-visceral), lines of metastatic systematic therapy of TLC (1 vs. 2 vs. ≥3), prior exposure to lapatinib were significantly correlated with PFS in Log rank analysis (Table 4). However, only lines of metastatic systematic therapy of TLC (1 vs. 2 vs. ≥3) proved to be an independent predictor of PFS in Cox multivariate analysis (Table 4). Though patients naïve to lapatinib had a significant longer PFS than those are not, prior of lapatinib was not an independent predicator PFS of TLC (Table 4, Figure 6).

**Table 4:** Log-rank and Cox multivariate analysis of factors associated with progression free survival.

**Characteristic**	**HR (95% CI)**	**Log-rank analysis *P*-value**	**HR (95% CI)**	**Cox multivariate analysis *P*-value**
Age group (<60 vs. ≥60)	1.174 (0.835–1.652)	0.356		
Hormone receptor status (HR+ vs. HR-)	1.062 (0.794–1.420)	0.684		
DFI (>1year vs. ≤1year)	1.181 (0.821–1.700)	0.370		0.328
Number of metastatic sites (≤2 vs. >2)	1.661 (1.245–2.216)	0.001	1.373 (0.995–1.895)	0.054
Types of metastasis (visceral vs. non-visceral)	1.511 (1.075–2.124)	0.018	1.079 (0.738–1.577)	0.650
Lines of advanced systematic therapy of TLC (1 vs. 2 vs. ≥3)	1.891(1.557–2.297)	<0.001	1.778(1.445–2.187)	<0.001
Trastuzumab Resistance Status (resistance vs. refractoriness)	1.244 (0.918–1.685)	0.159		
Prior exposure to lapatinib (yes vs. no)	1.684(1.179–2.405)	0.005	1.257 (0.868–1.819)	0.302

*HR, Hormone receptor; DFI, Disease free interval; TLC, trastuzumab+ lapatinib+ chemotherapy*.

### Safety

As we collected AEs based on patients' laboratory tests results and medical records, and given the retrospective nature of the study, omission of AEs was unavoidable. Here we report the grade 3 to 4 AEs (Table 5). The most common grade 3 and 4 AEs were neutropenia (16.8%), leukopenia (14.7%), diarrhea (9.5%), palmar–plantar erythrodysesthesia syndrome (PPES, 8.4%), anemia (7.7%). No treatment-related death was reported during TLC. In patients received initial TL plus capecitabine, the most common grade 3 to 4 AEs were PPES (12.9%) and diarrhea (6.9%). Overall, the safety of TLC was controllable and tolerable.

**Table 5:** Grade 3 to 4 adverse events.

**Grade 3 to 4 adverse events**	**Number of patients (%) (*N* = 285)**
Neutropenia	48 (16.8)
Leukopenia	42 (14.7)
Diarrhea	27 (9.5)
PPES	24 (8.4)
Anemia	22 (7.7)
Rash	7 (2.5)
Thrombocytopenia	6 (2.1)
Febrile neutropaenia	3 (1.1)
Infection	2 (0.7)
Vomit	2 (0.7)
Mucositis oral	2 (0.7)
Blood bilirubin increased	1 (0.7)
Fatigue	1 (0.4)

*PPES, Palmar-plantar erythrodysesthesia syndrome*.

## Discussion

Pertuzumab plus trastuzumab in combination with a taxane has become a preferred option for first-line treatment of patients with HER2+ MBC, and T-DM1 has become the preferred regimen for patient's progression after treatment with trastuzumab-based therapy. Regrettably, due to drug availability, pertuzumab, T-DM1 was not available in China, resulting the lack of choices of these anti-HER2 drugs in our clinical practice. Thus, the most common anti-HER2 agents we used to be trastuzumab and lapatinib. In clinical trials, the combination of TL has been shown to improve outcomes in both the neoadjuvant and metastatic settings. In order to further improve the efficacy, adding chemotherapy to TL has been commonly used in routine clinical practice. However, the efficacy and safety of TLC is still lack of evidence, especially in metastatic setting.

Though previous study had reported the efficacy and safety of TL in real-world (17), study of combined chemotherapy based on TL have not been reported. As far as we know, this is the first large-scale, retrospective, multicenter report of triple combination of TLC in real-world setting. The aim of our study was to investigate the efficacy and safety of TLC treatment and to explore the treatment pattern of this regimen.

In our study, considering patients' compliance, economic conditions and quality of life, 93 patients were treated with initial combination chemotherapy for 6–8 cycles, and then maintenance therapy with oral drugs, such as endocrine therapy and so on. Nearly half of the patients received lapatinib at an initial dose of 1,250 mg/day, perhaps out of the consideration for intolerable toxicity which may come along with higher dose of lapatinib when combined with trastuzumab and chemotherapy. Despite heavily pretreated patient population and compromise dosage of lapatinib, our study showed promising effects of TLC with a median PFS of 10.9 months and an ORR of 42.6%.

Median PFS was 20.7 months in our patients received TLC as first-line treatment, comparable with the results of trastuzumab, pertuzumab plus docetaxel reported in CELEOPATRA study (18.7 months) (12). Comparing patients characteristics between two populations, we could find less patients with visceral metastases (67.7% vs. 78.1%) and more patients with prior trastuzumab treatment (56.9% vs. 11.7%) and BM (24.7% vs. 0%) in our first-line patients than in CELEOPATRA study. Puffin study, as the bridge study of cleopatra study in China, showed the median PFS of 14.5 months in dual HER2 blockages group in Chinese patients (18). RePer study explored the efficacy of first-line treatment with pertuzumab/trastuzumab/taxane in real-world setting, demonstrated a median PFS s of 21 months (19). Our study showed a similar median PFS of 20.7 months in first-line, suggested that TLC had great potential as first-line treatment.

Based on the results of EGF100151, LX has become the standard regimen for patient's progression on trastuzumab and widely used in clinical practice. Almost 40% patients in our study received TL plus capecitabine (TLX), thus we explored that benefit of continuing trastuzumab in the basis of LX treatment in patients progressed on trastuzumab. Still, the triplet had achieved an ORR of 30.9% (Supplemental Table 3) and the median PFS of 11.3 months, numerically higher than that of LX reported in EFG100151 (22% and 8.4 months, respectively), indicating a continuing anti-tumor effect of trastuzumab. Patients characteristics were comparable between two studies. However, our study included 12% patients who had received prior lapatinib, whereas prior lapatinib was not allowed in EGF100151. In terms of safety, compared to EGF100151, less patients experienced grade 3 to 4 diarrhea (7.5% vs. 13.0%), which might due to a relatively lower dose of lapatinib applied in our study, while more patients suffered from grade 3 to 4 PPES (11.2% vs. 7.0%) in our analysis.

No grade 3/4 cardiac toxicity was observed, which indicated the cardiac safety of TLC regimen. Though the direct comparison was not inappropriate, our analysis suggested that continued use of trastuzumab in the basis of standard LX treatment among patients had progression on trastuzumab may provide benefit.

LACOG 0801 study investigated the efficacy and safety of lapatinib combined with capecitabine (LX), vinorelbine (V), or gemcitabine (G) in patients with HER2+ MBC with progression after a taxane (20). The results show that LX has similar efficacy with the other 2 arms, though LV has a more numerically longer median PFS and OS (20). Our results also showed that TL combined with capecitabine had a numerically longer median PFS than vinorelbine, similar to previously reported in LACOG0801 study, suggesting that the two regimens seem to be active combinations in HER2+ MBC.

Patients with HER2+ MBC have an increased risk of developing BM, comparing with HER2 negative patients. For patients with BM, treatments are limited and prognosis remains poor. Lapatinib, due to its “small molecular size and a high blood brain barrier (BBB) penetrability” (21), has become an important treatment strategy for these patients. In LANSCAPE trial, LX showed the high ORR of 65.9% and “a 5.5-month median time to progression” in not heavily pretreated HER2+ MBC with BM (22). A pooled analysis including 12 studies demonstrated that LX achieved an ORR of 30% and the median pooled PFS of 4 months in HER2+MBC with BM (23). Trastuzumab, due to the fact that its large-molecule property hinders it readily through BBB, was considered to have poor effects in BM. However, regist HER (24, 25), a prospective, observational study showed that trastuzumab treatment after BM was associated with longer survival. A large retrospective, multicenter study which enrolled 432 HER2+ patients diagnosed with BM demonstrated that patients received TL after developing metastasis significantly prolonged survival than patients treated with T alone, L alone, or no HER2-targeting regimen (*p* < 0.001) (24). In addition, Trastuzumab treatment after diagnosis of BM could reduce the risk of death (24). Another retrospective study of patients with BM reported the similar results (25). Patients received TL had a longest OS than those received L alone, T alone, and no anti-HER2 regimen “(25.9, 21.4, 10.5, and 5.7 months, respectively, *p* < 0.001)” (25). Our study showed a median PFS (intracranial and extracranial lesions considered) of 10.6 months, which is numerically higher or at least comparable with afore hand studies. All these data suggest great potential of TLC in controlling BM. Considering the small sample number of BM patients, conclusion should be drawn more carefully before more evidence from large size clinical trials.

The additive effects of the triplet combination bring the increased incidence of AEs, but in general, the combinational treatment was tolerable. Incidence of grade 3 and 4 diarrhea was lower than previously studies of LX, which may be due to the relatively low doses of lapatinib used in our study (only 49.5% of patients received lapatinib at an initial dose of 1,250 mg/day). In addition, due to the retrospective nature, oblivion in AEs was unavoidable.

It was a retrospective and observational study; therefore, it is limited to include potential missing data, possible recall and information bias. Besides, it has been difficult to perform dose-effect analyses due to the complexity of treatment pattern and retrospective nature of this study. Furthermore, the length of follow up was relatively short and overall insufficient to draw the OS conclusions. Our study also has some relevant strengths, since it provides evidence in support of the activity of the triplet combination of TLC in real-world practice and, to our knowledge, it is the first and largest observational case series made available thus far. Moreover, our results report the treatment pattern and safety data of TLC in clinical practice, providing a theoretical basis for clinical physician.

## Conclusion

To conclude, the triplet combination of trastuzumab, lapatinib and chemotherapy demonstrated promising efficacy in HER2+ MBC with tolerable toxicity. In patients with BM, TLC also demonstrated promising anti-tumoral activity. More clinical trials are needed to further exploit the potential of triplet combination.

## Data Availability Statement

All datasets analyzed for this study are included in the article/Supplementary Material.

## Ethics Statement

The studies involving human participants were reviewed and approved by Fudan University Shanghai Cancer Center. The patients/participants provided their written informed consent to participate in this study. Written informed consent was obtained from the individual(s) for the publication of any potentially identifiable images or data included in this article.

## Author Contributions

YL, BW, FX, RG, and ZZ conceived and designed the study. YL, CG, QL, ZZ, TL, WL, and GL collected the data. YL, CG, and QL performed the statistical analyses. YL and CG wrote the manuscript. BW, FX, and RG revised the manuscript. All authors approved the final manuscript.

### Conflict of Interest

The authors declare that the research was conducted in the absence of any commercial or financial relationships that could be construed as a potential conflict of interest.
