# Supplementary material for: Real-World Data of Triplet Combination of Trastuzumab, Lapatinib, and Chemotherapy in HER2-Positive Metastatic Breast Cancer: A Multicenter Retrospective Study
Source: Front Oncol. 2020 Mar 3;10:271. doi: 10.3389/fonc.2020.00271 (PMC7062863; doi:10.3389/fonc.2020.00271)
Supplement: Supplementary file 1 [file Data_Sheet_1.docx]

Supplemental Table 1. Baseline characteristics of patient received TLC as first-line treatment.

| Characteristics | Number of patients (%)  (N=65) |
| --- | --- |
| Median age (years, range) | 49 (26-73) |
| ECOG performance-status score  0  1  2 | 35 (53.8)  30 (46.2)  0 (0.0) |
| Hormone receptor status  HR positive  HR negative | 21 (32.3)  44 (67.7) |
| Disease-free interval  Primary metastatic  DFI≤1year  DFI>1year | 14 (21.5)  21 (32.3)  30 (46.2) |
| Metastatic sites  Lung  Liver  Bone  Brain | 20 (30.8)  13 (20.0)  17 (26.2)  16 (24.6) |
| Number of metastatic sites  1  2  ≥3 | 34 (52.3)  16 (24.6)  15 (23.1) |
| Visceral metastases  Yes  No | 44 (67.7)  21 (32.3） |
| Trastuzumab Resistance Status  Resistance  Refractoriness  Unknown | 11 (17.0)  29 (44.6)  25 (38.5) |
| Prior HER2-targeted therapy  Trastuzumab  Lapatinib  T-DM1  Pertuzumab | 37 (56.9)  0 (0.0)  0 (0.0)  0 (0.0) |

Abbreviations: HR, Hormone receptor; ECOG, Eastern Cooperative Oncology Group; DFI, Disease free interval; TLC, trastuzumab+ lapatinib+ chemotherapy

Supplemental Table 2. Baseline characteristics of patient received TLX after progressing on trastuzumab.

| Characteristics | Number of patients (%)  (N=110) |
| --- | --- |
| Median age (years, range) | 52.5(29-87) |
| ECOG performance-status score  0  1  2 | 39 (35.5)  66 (60.0)  5 (4.5) |
| Hormone receptor status  HR positive  HR negative | 44 (40.0)  66 (60.0) |
| Disease-free interval  Primary metastatic  DFI≤1year  DFI>1year | 26 (23.6)  19 (17.3)  65 (59.1) |
| Metastatic sites  Lung  Liver  Bone  Brain | 38 (34.5)  40 (36.4)  49 (44.5)  26 (23.6) |
| Number of metastatic sites  1  2  ≥3 | 28 (25.5)  36 (32.7)  46 (41.8) |
| Visceral metastases  Yes  No | 80 (72.7)  30 (27.2) |
| Lines of advanced systematic therapy of TLC  1  2  ≥3 | 19 (17.3)  41 (37.3)  50 (45.5) |
| Trastuzumab Resistance Status  Resistance  Refractoriness | 45 (40.9)  65 (59.1) |
| Prior HER2-targeted therapy  Trastuzumab  Lapatinib  T-DM1  Pertuzumab | 110 (100.0)  13 (11.8)  1 (0.9)  0 (0.0) |

Abbreviations: HR, Hormone receptor; ECOG, Eastern Cooperative Oncology Group; DFI, Disease free interval; TLX, trastuzumab+ lapatinib+ capecitabine

Supplemental Table 3. Objective response rate in patients received TLX.

| Response | Number of patients (%)  (N=110) |
| --- | --- |
| Best response  Complete response  Partial response  Stable disease  Progressive disease  NA  ORR | 0 (0.0)  34 (30.9)  64 (58.2)  8 (7.3)  4 (3.6)  34 (30.9) |

Abbreviation: NA, not available; ORR: objective response rate; TLC, trastuzumab+ lapatinib+ capecitabine
